# Clinical evaluation of 4D MRI in the delineation of gross and internal tumor volumes in comparison with 4DCT

**DOI:** 10.1002/acm2.12699

**Published:** 2019-09-20

**Authors:** Jingjing Zhang, Shreya Srivastava, Chunyu Wang, Thomas Beckham, Christopher Johnson, Pinaki Dutta, Annemarie Shepherd, James Mechalakos, Margie Hunt, Abraham Wu, Andreas Rimner, Guang Li

**Affiliations:** ^1^ Department of Radiation Oncology Zhongshan Hospital of Sun Yat‐Sen University Zhongshan China; ^2^ Department of Medical Physics Memorial Sloan Kettering Cancer Center New York NY 10065 USA; ^3^ Department of Radiation Oncology Memorial Sloan Kettering Cancer Center New York NY 10065 USA

**Keywords:** magnetic resonance imaging, motion artifacts, respiratory motion simulation, treatment planning, tumor delineation

## Abstract

**Purpose:**

To evaluate clinical utility of respiratory‐correlated (RC) four‐dimensional magnetic resonance imaging (4DMRI) for lung tumor delineation and motion assessment, in comparison with the current clinical standard of 4D computed tomography (4DCT).

**Methods and Materials:**

A prospective T2‐weighted (T2w) RC‐4DMRI technique was applied to acquire coronal 4DMRI images for 14 lung cancer patients (16 lesions) during free breathing (FB) under an IRB‐approved protocol, together with a breath‐hold (BH) T1w 3DMRI and axial 4DMRI. Clinical simulation CT and 4DCT were acquired within 2 h. An internal navigator was applied to trigger amplitude‐binned 4DMRI acquisition whereas a bellows or real‐time position management (RPM) was used in the 4DCT reconstruction. Six radiation oncologists manually delineated the gross and internal tumor volumes (GTV and ITV) in 399 3D images using programmed clinical workflows under a tumor delineation guideline. The ITV was the union of GTVs within the breathing cycle without margin. Average GTV and motion range were assessed and ITV variation between 4DMRI and 4DCT was evaluated using the Dice similarity index, mean distance agreement (MDA), and volume difference.

**Results:**

The mean tumor volume is similar between 4DCT (GTV^4DCT^ = 1.0, as the reference) and T2w‐4DMRI (GTV^T2wMR^ = 0.97), but smaller in T1w MRI (GTV^T1wMR^ = 0.76), suggesting possible peripheral edema around the tumor. Average GTV variation within the breathing cycle (22%) in 4DMRI is slightly greater than 4DCT (17%). GTV motion variation (−4 to 12 mm) and ITV variation (∆V^ITV^=−25 to 95%) between 4DCT and 4DMRI are large, confirmed by relatively low ITV similarity (Dice = 0.72 ± 0.11) and large MDA = 2.9 ± 1.5 mm.

**Conclusion:**

Average GTVs are similar between T2w‐4DMRI and 4DCT, but smaller by 25% in T1w BH MRI. Physician training and breathing coaching may be necessary to reduce ITV variability between 4DMRI and 4DCT. Four‐dimensional magnetic resonance imaging is a promising and viable technique for clinical lung tumor delineation and motion assessment.

## 1 INTRODUCTION

Respiratory‐correlated four‐dimensional magnetic resonance imaging (4DMRI) provides patient‐specific respiratory motion with high soft‐tissue contrast without ionizing radiation, in comparison with 4D computed tomography (4DCT), the current clinical standard in lung tumor motion assessment.^1^ In addition, 4DMRI allows utilizing an internal navigator as a respiratory surrogate, eliminating the uncertainty from an assumed external–internal motion correlation of an external surrogate used in the 4DCT acquisition. Thus, a navigator‐triggered/binned 4DMRI has higher image quality with fewer and less severe binning artifacts.^2^, ^3^, ^4^, ^5^, ^6^ Furthermore, MRI provides the option of the nonaxial scanning direction, such as sagittal or coronal scans, which are more desirable for characterizing tumor/organ respiratory motion.^7^, ^8^ Therefore, 4DMRI promises to be clinically beneficial in assessing respiratory‐induced tumor motion.^9^, ^10^, ^11^

Although normal lung has low MR signal from the “air‐diluted” soft tissue, a lung tumor usually has higher density and produces sufficient MR signal, including lung tumor perfusion with dynamic contrast enhancement imaging^12^, ^13^ and lung tumor microenvironment with diffusion‐weighted imaging.^14^, ^15^ In lung tumor motion assessment and monitoring, dynamic two‐dimensional (2D) cine imaging has been widely applied, including MR‐guided radiotherapy,^7^, ^16^, ^17^, ^18^, ^19^, ^20^ automatic tumor contouring for motion tracking,^21^, ^22^ and tumor motion variation during radiotherapy.^23^, ^24^, ^25^ A fast field echo with either balanced steady‐state free precession or T1‐weighted (T1w) 2D cine has been used to achieve 4 Hz frame rate.^7^, ^16^, ^17^, ^18^, ^19^, ^20^

For treatment planning purposes, volumetric 4DMRI is required so that both lung tumor and surrounding normal organs can be delineated for accurate targeting and motion assessment, using the gross and internal tumor volume (GTV and ITV). Recently, 4DMRI has been assessed for delineating five organs and propagating the contours between different respiratory states.^26^ Among various MR contrast, T2‐weighted (T2w) 4DMRI provides higher tissue contrast for GTV delineation^4^, ^27^ and the clinical utility needs to be further assessed in comparison with 4DCT.

In this study, we present the comparison of lung tumor delineation based on T2w 4DMRI, T1w BH MRI, and 4DCT by six radiation oncologists in 14 lung cancer patients with 16 lesions, which were grouped by location (central vs peripheral) and size (small, medium, and large). The comparison includes GTV variation within a breathing cycle and average GTV difference among these imaging modalities. Furthermore, GTV motion variation was assessed and ITV difference between 4DMRI and 4DCT was characterized in terms of size and shape. The clinical implication of the lung tumor delineation using 4DMRI and 4DCT was discussed.

## 2 METHODS AND MATERIALS

An IRB‐approved protocol was established and 14 lung cancer patients were scanned using a 3 T MRI scanner (Φ = 70 cm, Ingenia, Philips Healthcare, the Netherlands) after clinical CT and 4DCT scans for treatment planning using a helical CT scanner (Φ = 85 cm, big‐bore brilliant, Philips Healthcare, the Netherlands) or a cine PET/CT scanner (Φ = 70 cm, Discovery, STE, GE Healthcare, Milwaukee, WI).

### 2.1 Acquisition of clinical 4DCT and planning CT

Clinical 4DCT and planning CT images were acquired first before MR scans, within 1–2 h on the same day. The patient body immobilization mold was prepared in the CT room and its width (<70 cm) was made to fit in the MR scanner. The patient was asked to have both arms up above the head and wear an MR headphone during molding for later MR scans.

Standard clinical thoracic CT/4DCT scan protocols were applied with a voxel size of 1 × 1 × 3 mm^3^ covering the entire lung. The planning CT was first acquired in free breathing, followed by the 4DCT scan. A bellows or real‐time position management device was placed around 5–10 cm inferior to the xiphoid process of the sternum as the respiratory surrogate for retrospective amplitude‐binned 4DCT reconstruction.

### 2.2 Image acquisition of T2w 4DMRI and T1w BH MRI

The MR scans were performed after CT scans using the same body mold. A prospective navigator‐triggered amplitude‐binned T2w 4DMRI scanning protocol was applied to acquire the 4D images with 2 × 2 × 5 mm^3^ voxel size in the coronal direction. The navigator is a dynamic 1D image (20 Hz) within a small field of view (3 × 3 × 6 cm^3^) set at the right diaphragm dome to detect internal motion signal (waveform) based on the image intensity gradient for respiratory binning. The first 10‐second navigator waveform was acquired and used to train the system for an amplitude triggering to fill the bin‐slice table (10 bins vs anterior–posterior slices). The pulse sequence was a single‐shot, turbo spin echo with TE/TR = 80/5000–7000 ms, flip angle = 90º; SENSE (SENSitivity Encoding) factor = 2, and a half‐scan factor = 0.7. Three‐to‐four segments were used to avoid signal saturation due to two consecutive acquisitions from the same segment. As control experiments, the axial 4DMRI scan (10 bins at 2 × 2 × 5 mm^3^) and high‐resolution coronal 4DMRI scan (3 bins at 2 × 2 × 2 mm^3^) were applied for first and last seven patients, respectively. By estimation, all 4DMRI scans would take a similar time range (5–15 min).^8^

A T1w turbo field echo (TFE) sequence was employed with TE/TR of 1.9 ms/4.2 ms and a flip angle of 15°. Parallel imaging (SENSE factor of 3), a half‐scan factor of 0.8, and central‐to‐peripheral k‐space acquisition order (CENTRA) were employed. The coronal direction with the smallest body separation (so least slice number) was used for acquisition, while the lateral direction with minimal motion was set for phase encoding. The same field of view for T2w 4DMRI was applied for T1w BH MRI with the voxel size of 2 × 2 × 2 mm^3^. More detailed scan parameters in 4DMRI and BH MRI were reported previously.^5^, ^28^

### 2.3 Manual Lung tumor delineation conditions and procedures

Six radiation oncologists manually delineated 16 lung tumors in 399 3D CT/MR images using two programmed MIM workflows (MIM software) for image loading, registration, and segmentation in 4DCT and 4DMRI. The 4D images were first automatically registered to the planning CT based on maximum mutual information, followed by GTV delineation in a selected respiratory‐state image that contained minimal binning artifacts. The GTV was propagated to other states automatically using B‐spline deformable image registration and corrected manually by the physicians. A guideline for manual lung tumor delineation was provided to specify visualization conditions, including a zoom factor and linear window/level (W/L): W=−1024–300 HU in 4DCT, W = 0–1200 in T2w 4DMRI, and W = 0–600 in T1w BH MRI. This was based on a visual assessment of the lung tumors in the CT/T2w/T1w images under various W/L to reach a steady tumor size in the imaging modalities, owing to the high tumor/lung contrast. For simplicity, only the primary GTV was delineated without considering nodal involvement. Only GTV was delineated in T1w BH MRI. The ITV was automatically calculated without a margin.

### 2.4 Analysis of multiple datasets of lung tumor contours

The 16 lung lesions were first categorized based on their location (central vs. peripheral), as the delineation precision of peripheral lesions should be higher than central lesions due to the well‐defined boundary. The lesions were then sorted by size, which also impacts on the contour uncertainty and tumor mobility. A small tumor has a volume of <10 cc, a medium tumor has 10–30 cc, and a large tumor has >30 cc.

Four aspects of the GTV/ITV delineation were analyzed. First, the average GTV was compared among 4DCT, T2w 4DMRI, and T1w MRI. Second, GTV variation was compared within the breathing cycle and between 4DMRI and 4DCT. Third, GTV displacement (center of mass, COM) was compared between 4DMRI and 4DCT. Fourth, the volume and shape of the ITV were compared between 4DMRI and 4DCT, after the alignment of ITVs based on their COM, using the Dice similarity index and mean distance agreement (MDA) for quantification.

Because of differences in viewing direction (axial CT vs coronal MRI) and image resolution (1 × 1 × 3 mm^3^ for CT/4DCT and 2 × 2 × 5 mm^3^ for 4DMRI), two sets of control experiments were performed. The first seven patients were also scanned with 4DMRI in axial view and the last seven patients also were scanned with a higher resolution of 2 × 2 × 2 mm^3^.

## 3 RESULTS

### 3.1 Average GTV and its variation from CT to T2w and T1w MRI

**Figure 1:**
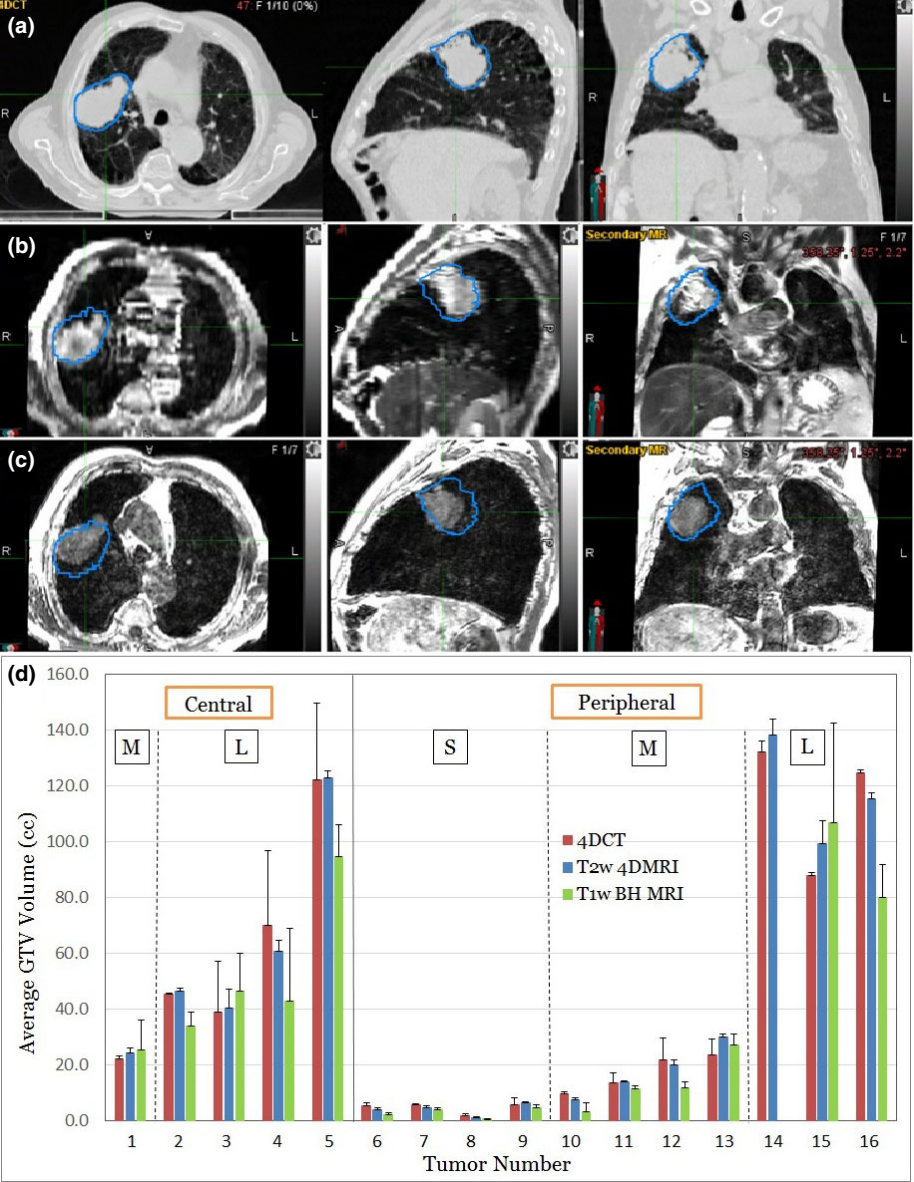
An example and statistics of lung tumor delineation. The GTV contour delineated in 4DCT (a) is superimposed to a similar respiratory state of T2w 4DMRI (b) and to the T1w BH MRI (c), and the GTV in 4DCT is greater than those in the T2w and T1w MRI images. A plot of all 16 delineated GTV in three modalities (d). The GTVs are sorted based on their location (central vs peripheral) and size (S: small, M: medium, L: large). 4DCT, 4D computed tomography; 4DMRI, four‐dimensional magnetic resonance imaging; BH, breath‐hold; GTV, gross tumor volumes.

All 16 lung tumors are visualized in 4DCT, T2w 4DMRI, and T1w BH MRI, although some noticeably different appearances are observed, as shown in Fig. 1. The average and standard deviation of GTV in 4DCT, T2w 4DMRI, and T1w BH MRI are shown in Fig. 1(d). For small and medium‐sized peripheral tumors, the tumor boundary is well defined except that it may contact the chest wall. The GTV is similar between 4DCT and T2w 4DMRI, while the GTV from T1w MRI is on average 24% smaller. A similar trend was found for all lesions: the average tumor volume ratios are GTV^T2w^/GTV^CT^ = 0.97 ± 0.16 and GTV^T1w^/GTV^CT^ = 0.76 ± 0.30, as shown in Table 1.

**Table 1:** Gross tumor volume (GTV, in cc) variation between 4DCT, T2w 4DMRI, and T1w breath‐hold (BH) MRI.

Tumor	Site#	Size$	4DCT		T2w 4DMRI		T1w BH MRI		GTV Ratio	
			Mean	St dev	Mean	St dev	Mean	St dev	T2w/CT	T1w/CT
1	C	M	22.26	0.92	24.38	1.74	25.39	10.74	1.10	1.14
2	C	L	45.30	0.47	46.40	1.10	34.10	5.00	1.02	0.75
3	C	L	39.00	18.24	40.22	6.81	46.30	13.62	1.03	1.19
4	C	L	69.85	26.93	60.82	3.74	42.97	25.88	0.87	0.62
5	C	L	121.97	27.51	122.65	2.76	94.72	11.24	1.01	0.78
6	P	S	5.32	1.28	4.08	0.70	2.06	0.90	0.77	0.39
7	P	S	5.97	0.23	4.89	0.44	4.07	0.58	0.82	0.68
8	P	S	2.03	0.66	1.33	0.29	0.72	0.25	0.66	0.36
9	P	S	5.96	2.47	6.49	0.34	4.58	1.33	1.09	0.77
10	P	M	9.74	0.53	7.68	0.58	3.48	2.95	0.79	0.36
11	P	M	13.60	3.61	13.85	0.60	11.48	1.19	1.02	0.84
12	P	M	21.88	7.64	20.10	1.78	11.84	2.06	0.92	0.54
13	P	M	23.70	5.63	29.96	1.25	27.10	4.06	1.26	1.14
14	P	L	131.94	3.94	138.13	5.67	–	–	1.05	–
15	P	L	87.83	1.00	99.14	8.44	106.83	35.69	1.13	1.22
16	P	L	124.59	1.05	115.29	1.97	79.93	11.75	0.93	0.64
Average			45.68	6.38	45.96	2.39	33.04	8.48	0.97	0.76
St Dev			46.55	9.29	47.01	2.51	35.11	10.31	0.16	0.30

4DCT, 4D computed tomography; 4DMRI, four‐dimensional magnetic resonance imaging; GTV, gross tumor volumes.

^#^ The lung tumor location is categorized as central (C) or peripheral (P).

^$^ The GTV size is small (<10 cc), medium (10–30 cc), and large (>30 cc).

### 3.2 Variation of GTV within the breathing cycle of 4DMRI and 4DCT

**Figure 2:**
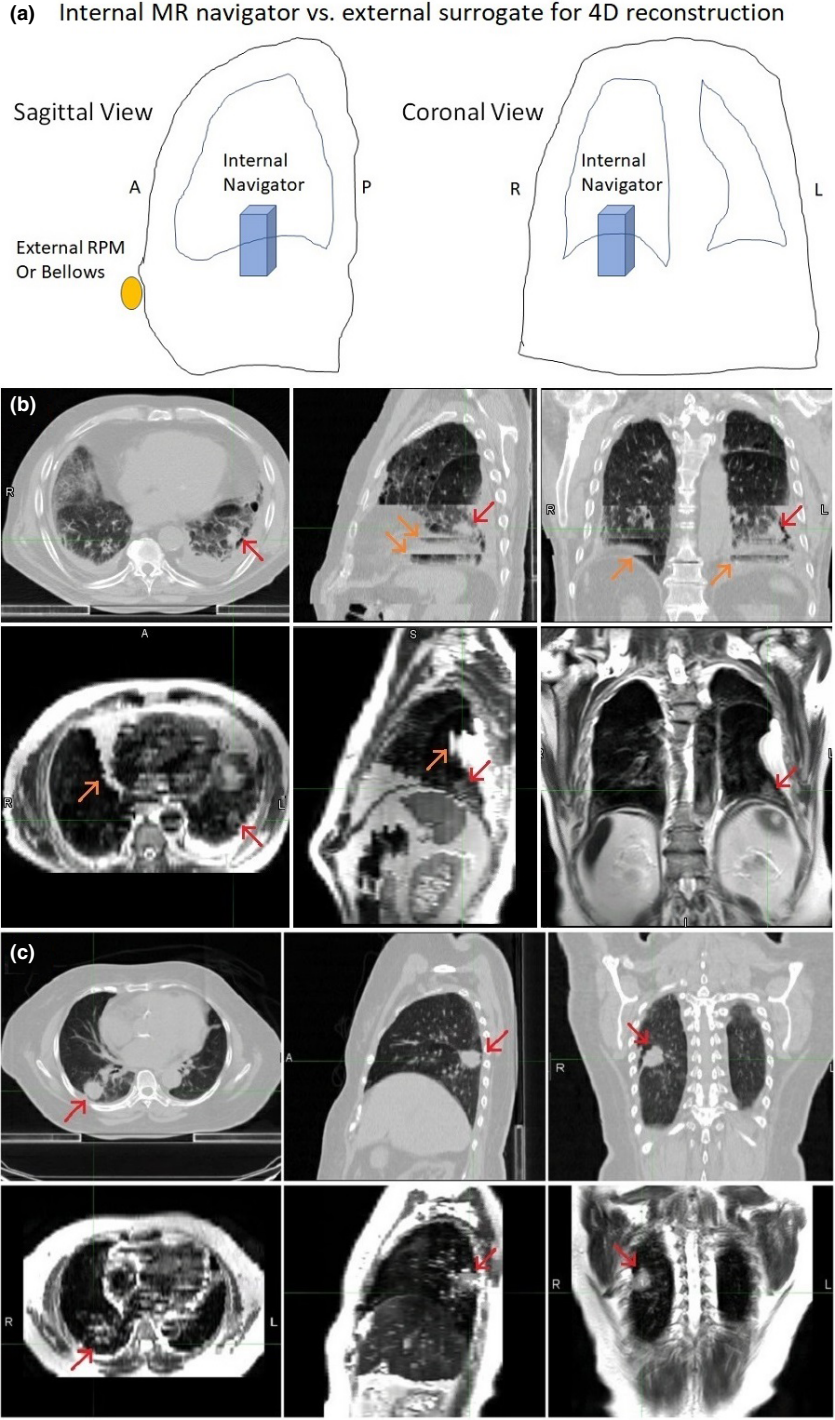
Internal and external respiratory motion surrogates and different image quality and tumor visibility in 4DCT (high‐resolution axial scan) and 4DMRI (low‐resolution coronal scan) of two small peripheral lesions. (a) a schematic drawing of the internal navigator and external real‐time position management (RPM) or bellows on a patient. (b) Tumor #6 (red arrow) with a large motion and large binning artifacts (orange arrows) in 4DCT and minor artifacts (orange arrow) in 4DMRI. (c) Tumor #11 (red arrow) with medium motion and mild artifacts in 4DCT and 4DMRI images. 4DCT, 4D computed tomography; 4DMRI, four‐dimensional magnetic resonance imaging.

The GTV variation within the breathing cycle may result from 4D image quality (artifacts) and intra‐observer variation. Figure 2 illustrated the image quality difference of 4DCT and 4DMRI of two patients and the difference would affect tumor delineation, especially smaller tumors with large motions. The mean variation of GTV within the breathing cycle among six radiation oncologists is slightly greater in 4DMRI (22%) than 4DCT (16%). The GTV ratios of axial T2w 4DMRI to 4DCT (0.96 ± 0.10) and high‐resolution coronal T2w 4DMRI to axial 4DCT (1.04 ± 0.13) are close to unity, similar to 0.97 ± 0.16 for low‐resolution T2w 4DMRI, suggesting that contouring directions and slice thickness difference are not critical in lung tumor delineation.

### 3.3 Variation of GTV motion between 4DMRI and 4DCT

**Figure 3:**
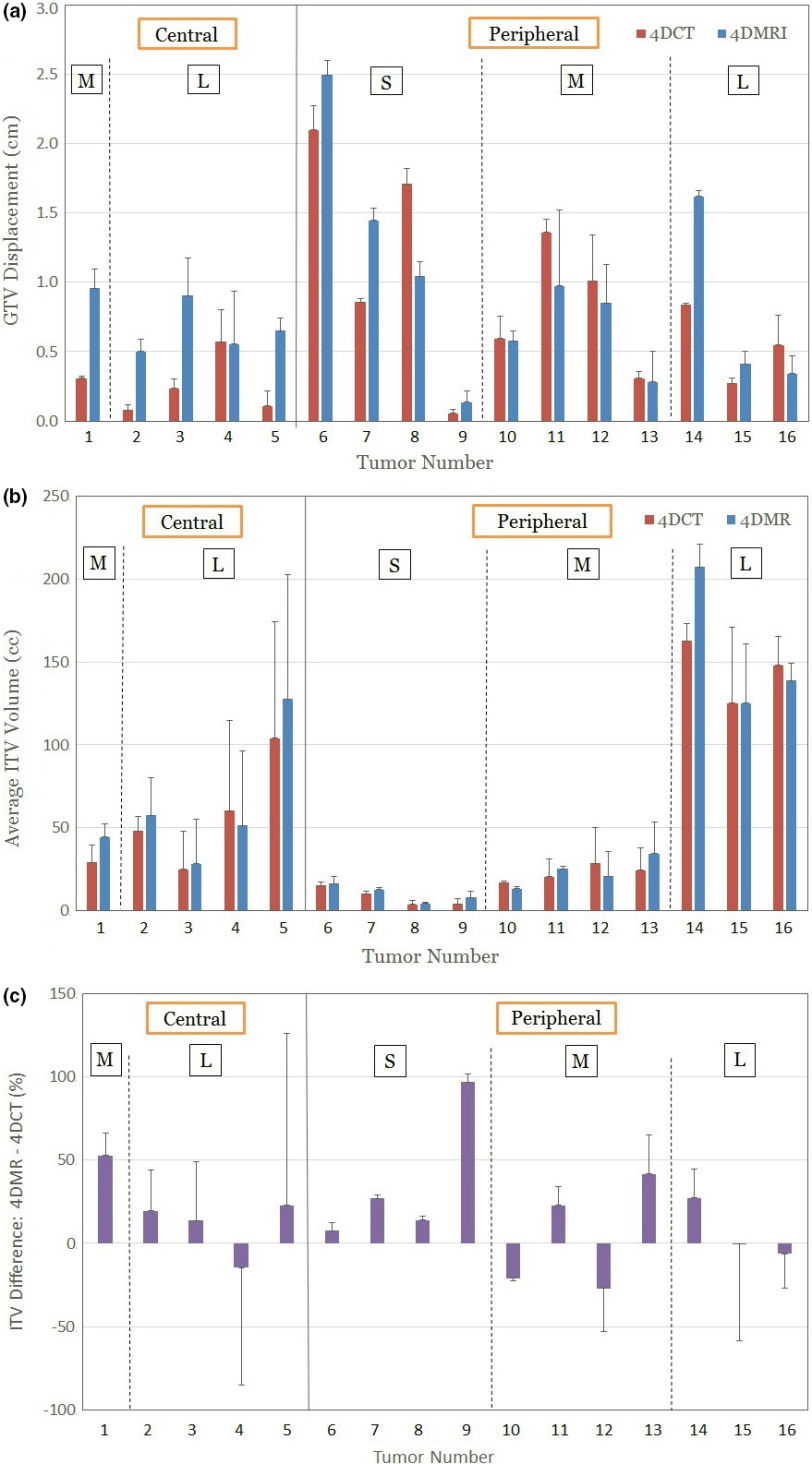
Mean GTV displacement difference (a) and ITV variation between 4DCT and 4DMRI (b and c). The GTVs are sorted based on their location (central vs peripheral) and size (S: small, M: medium, L: large). The error bars (1σ) are from GTV and ITV delineation by the six physicians. Five out of 16 lesions (~31%) have motion variation >5 mm and mean ITV varies from −25% to + 95% between 4DCT and 4DMRI. 4DCT, 4D computed tomography; 4DMRI, four‐dimensional magnetic resonance imaging; GTV and ITV, gross and internal tumor volumes.

Tumor motion displacement varies between 4DCT and 4DMRI owing to patient breathing irregularities, as shown in Fig. 3(a). Five out of 16 tumors (~31%) have a displacement differing by >5 mm between 4DCT and 4DMR. In ~ 75% cases, the tumor moves similar or greater in 4DMRI than in 4DCT. It is worthwhile to mention that only the first 10‐s (2–3 cycles) FB motion waveform is used to determine the 4DMRI motion amplitude in the reconstructed image.

### 3.4 The difference of ITV size and shape between 4DMRI and 4DCT

Although ITV delineation is affected by both GTV motion and GTV delineation, the GTV motion difference plays a more significant role, especially for small mobile tumors. In this study, the union of all GTVs is regarded as the ITV without an extra margin. Fig. 3(b) shows the average ITV differences between 4DCT and 4DMRI, together with the variation from the six physicians. The relative ITV difference [(ITV^4DMRI^–ITV^4DCT^)/ITV^4DCT^ × 100%] is 16 ± 31%, as shown in Fig. 3(c). This is a substantial difference in target volume for treatment planning.

**Figure 4:**
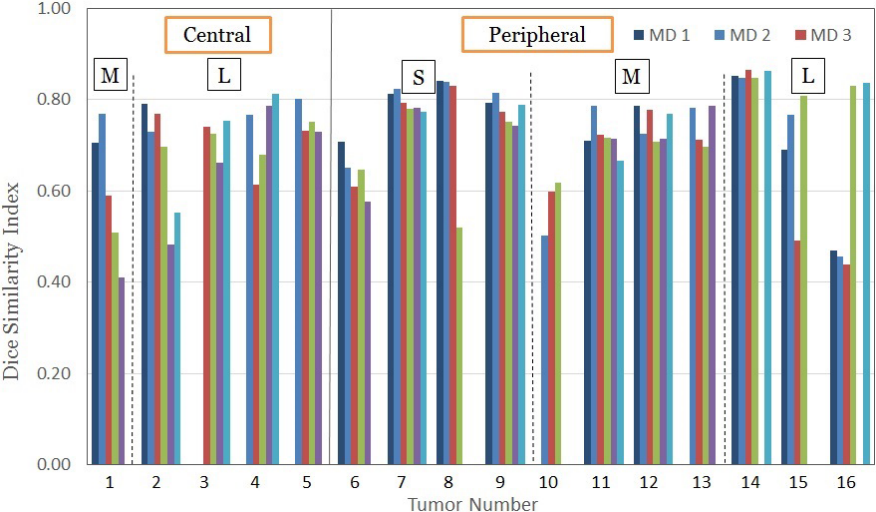
The Dice similarity index of the ITV between 4DCT and 4DMRI among six radiation oncologists (some tumors have incomplete datasets depending on the availability of physicians to delineate). The GTVs are sorted based on their location (central vs peripheral) and size (S: small, M: medium, L: large). Due to the GTV displacement and delineation difference, the ITV dice index varies from 0.41 to 0.87. 4DCT, 4D computed tomography; 4DMRI, four‐dimensional magnetic resonance imaging; GTV and ITV, gross and internal tumor volumes.

Table 2 tabulates the MDA and Dice similarity index of the ITV delineated between 4DCT and 4DMRI by six physicians. The mean MDA is 2.9 ± 1.5 mm (range: 0.9–9.0 mm), while the Dice index is 0.72 ± 0.11 (range: 0.41–0.86). When the Dice index is greater than 0.7–0.8, the MDA is usually 1.0–3.0 mm. Figure 4 illustrates the variation of the dice indices from the six radiation oncologists, suggesting that the large ITV variation is also largely associated with inter‐observer variation, in addition to tumor motion variation between 4DCT and 4DMRI scans. To our best knowledge, this is the first study that compares the GTV and ITV delineation between 4DMRI and 4DCT.

**Table 2:** ITV difference between 4DMRI and 4DCT quantified by the mean distance to agreement (MDA, mm) and Dice similarity index among six radiation oncologists. The site refers to central (C) or peripheral (P) and size refers to small (S: <10cc), medium (M: 10–30 cc), and large (L: >30 cc).

Tumor	Site	Size	MD1		MD2		MD3		MD4			MD5			MD6	
			MDA	Dice	MDA	Dice	MDA	Dice	MDA	Dice	MDA		Dice	MDA		Dice
1	C	M	2.4	0.70	2.5	0.77	4.3	0.59	4.7	0.51	5.7		0.41	–		–
2	C	L	2.1	0.79	2.7	0.73	2.4	0.77	3.1	0.70	7.8		0.48	8.3		0.55
3	C	L	–	–	–	–	2.5	0.74	2.9	0.72	4.1		0.66	3.1		0.75
4	C	L	–	–	3.1	0.77	5.7	0.60	4.2	0.68	2.7		0.79	2.3		0.81
5	C	L	–	–	2.6	0.80	4.3	0.73	3.4	0.75	6.3		0.73	–		–
6	P	S	2.0	0.71	2.4	0.65	2.9	0.61	2.4	0.65	5.0		0.58	–		–
7	P	S	1.3	0.81	1.2	0.82	1.4	0.79	1.4	0.78	1.5		0.78	1.7		0.77
8	P	S	2.3	0.84	2.6	0.84	2.5	0.83	2.2	0.52	–		–	–		–
9	P	M	0.9	0.79	0.9	0.81	1.1	0.77	1.2	0.75	1.3		0.74	1.3		0.79
10	P	M	–	–	3.7	0.50	2.5	0.60	2.3	0.62	–		–	–		–
11	P	M	2.6	0.71	1.8	0.79	2.3	0.72	2.3	0.72	2.5		0.72	3.5		0.67
12	P	M	1.6	0.79	2.8	0.72	1.9	0.78	3.3	0.71	2.7		0.71	2.4		0.77
13	P	M	–	–	2.1	0.78	2.6	0.71	2.9	0.70	1.9		0.79	–		–
14	P	L	2.5	0.85	2.7	0.85	2.3	0.87	2.7	0.85	–		–	2.6		0.86
15	P	L	2.5	0.69	2.8	0.77	9.0	0.49	2.7	0.81	–		–	–		–
16	P	L	3.1	0.47	2.9	0.46	2.7	0.44	2.3	0.83	–		–	2.5		0.84
Mean			2.12	0.74	2.44	0.74	3.15	0.69	2.75	0.71	3.78		0.67	3.07		0.76
St Dev			0.62	0.11	0.72	0.12	1.92	0.12	0.88	0.10	2.17		0.13	2.07		0.09

4DCT, 4D computed tomography; 4DMRI, four‐dimensional magnetic resonance imaging.

## 4 DISCUSSION

### 4.1 Lung tumor visualization and variation between imaging modalities

Although there is relatively low signal‐to‐noise ratio in lung MR imaging, T2w 4DMRI and T1w BH images provide sufficient visualization for tumor delineation and motion assessment for radiotherapy simulation, making it a potential alternative to 4DCT in the paradigm of MR‐guided radiotherapy. As an internal navigator can be used in 4DMRI acquisition for respiratory binning, the number of severe binning artifacts is substantially reduced and only minor artifacts remain,^28^ as shown in Figs. 1 and 2. However, 4DMRI carries a unique artifact that appears similar to binning artifacts but only in the heart, aorta/vena, and major artery/veins, because of blood flow that may move the excited protons away from the acquisition slice, depending on the flowrate, flow direction, and slice thickness. Additionally, the coronal scan preserves more anatomic integrity of superior–inferior motion. Although the image resolution of 4DMRI differs from 4DCT, the larger slice thickness (5 mm) in 4DMRI, which may affect tumor visualization, does not produce much difference in tumor delineation as the in‐slice resolution (pixel size of 2 × 2 mm^2^) seems quite acceptable. In fact, for tumor motion assessment, the coronal 4DMRI slices provide a 2.0 mm resolution, superior to 3 mm resolution in the superior–inferior direction of 4DCT. It is worthwhile to mention that when the tumor is small and motion is large, such as tumor #8 in Table 1, the voxel size and severity of the binning artifacts could have substantial impact on the delineation. This study has demonstrated that the GTV and ITV delineation from T2w 4DMRI is comparable with that from 4DCT.

Based on over 2000 GTV contours (16 lesions, three modalities, 10 bins, and six physicians) in various locations, sizes, and shapes, the average GTV difference is small (3%) between 4DCT and T2w 4DMRI (T2w‐to‐CT volume ratio is 97 ± 16%). However, GTV decreases by 24% from CT to T1w MRI (T1w‐to‐CT volume ratio is 0.76 ± 0.30). A hypothetical explanation is that lung lesions may have a thin layer of edema, which can be well visualized by both CT and T2w MRI but may not by T1w MRI. Interestingly, GTV from CT was reported to be 18.3% greater than that of the pathological specimen based on 47 stage I or II lung cancer patients,^29^ supporting this hypothesis. Another study based on 52 lung cancer patients illustrated that CT‐based GTV delineation is larger than integrated PET/CT‐based GTV, which was closer to that obtained from the pathological specimen.^30^ Although this edema hypothesis may be plausible, further investigation is necessary to provide direct evidence for support.

Between central and peripheral lesions, the major uncertainty in GTV delineation is from the border visualization of the gross tumor. The peripheral lesions often have clearly defined edge, and therefore the delineated GTV is more accurate than central lesions, which are likely attached to a local normal structure in the hilum, making the delineation of the GTV more subjective. Therefore, it is more challenging to delineate a centrally located lung lesion than a peripheral one. Although T2w 4DMRI provides better soft‐tissue contrast to differentiate the tumor from the surrounding central lung tissues, unlike 4DCT, further study and training are necessary to take advantage of 4DMRI. In this study, the inconsistency in determining the boundary of the GTV results in a large variation of the GTV delineation.

### 4.2 Inter‐observer variation in GTV and ITV delineation

The GTV delineation variation among the six radiation oncologists is consistently smaller in 4DCT (16%) than in T2w 4DMRI (22%), by 6% on average. One of the major possible reasons is related to insufficient physician training on using MRI for tumor delineation, as radiation oncologists are all trained and practiced with CT‐based tumor delineation. In this study, an image visualization guideline was used, such as window/level settings for MR images, aiming to minimize the inter‐observer variation. However, it seems not sufficient, as large intra‐ and inter‐observer variations in GTV delineation are observed. Therefore, more training of MR‐based tumor delineation seems necessary to reduce variations in GTV.

In addition to image modality difference, other differences in contouring lung tumors come from different viewing direction (axial CT view vs. coronal MR view) and the image resolution difference (1 × 1 × 3 mm^3^ vs 2 × 2 × 5 mm^3^). Although these may cause visualization differences, this study has shown minimal impact on GTV delineation based on two control comparisons: axial vs. coronal and high‐resolution vs. low‐resolution, using the extra T2w 4DMRI scans. In fact, the advantages of the coronal scans in 4DMRI are the integrity of the moving anatomy, the in‐slice motion has a higher spatial resolution, and^3^ the faster acquisition due to fewer slices in the AP direction. The GTV difference depends upon image quality as well as the experience of the users using both 4DCT and 4DMRI.

In this study, the inter‐observer variation is high, as indicated in high standard deviation (σ) in Table 1. The relative variation [(σ/mean) × 100%] on average decreases from T1w BH MR (25%), 4DCT (14%), and T2w 4DMRI (5%). In addition, the inter‐/intra‐observer variability increases when a tumor is attached to high‐intensity tissue, such as the chest wall or central non‐lung tissue. In fact, there are three such instances where the GTV in larger in T1w BH MRI than T2w 4DMRI, which is against the general trend we observed in this study, as shown in Table 1. Proper interpretation of tumorous and normal tissue needs improvement. It is also worthwhile to mention that the geometric distortion of the MR scanner is corrected using a large grid phantom. Within 35 cm region of interest around the isocenter, the residual distortion is about 1mm. Therefore, comparing with the inter‐/intra‐observer variation in manual tumor delineation, the MR distortion factor is negligible.

### 4.3 Tumor motion and ITV difference caused by breathing irregularities

Breathing irregularities may change the displacement of a mobile tumor within the breathing cycle during the 4DCT or 4DMRI scans. In 4DCT, tumor motion is determined within a few bed positions or helical pitches when scanning around the tumor within the field of view in a patient. So, it represents a composite tumor motion within the few breathing cycles. In 4DMRI, the first 10 s (2–3 cycles) determine the breathing amplitude of 4DMRI scan. Therefore, the ITV drawn based on either of the 4D images may not be truly representing tumor motion in a longer time frame during treatment. This study has illustrated significant tumor motion variation between 4DCT and 4DMRI, which may impact ITV by up to 100%, as shown in Fig. 3(c). It is worthwhile to indicate that this large ITV difference is caused by patient breathing irregularities rather than imaging modalities and both are correctly reflecting the ITV at the moment of scanning, but they may both deviate from the mean value if multi‐breath respiration motion is scanned and used to delineate the ITV, closer to the mean ITV value for treatment planning.

An alternative to the single‐breath 4DCT and 4DMRI is the multibreath volumetric time‐resolved 4DMRI, which has been reported lately to provide multiple breathing cycles over a time scale of minutes, rather than seconds.^28^, ^31^ Using the time‐resolved 4DMRI, patient‐specific multi‐breath tumor or organ motion can be better characterized and potentially incorporated into treatment planning and delivery for motion‐compensated radiotherapy.^32^, ^33^, ^34^

In summary, this study is the first attempt to compare lung tumor delineation between T2w 4DMRI and 4DCT. The additional image contrast provided by T2w 4DMRI may help to reduce the uncertainty in delineating centrally located tumors. However, given the limited clinical utility of MRI in current thoracic radiotherapy planning, additional studies, as well as training, will be needed for physicians to appropriately interpret the soft‐tissue contrast in MRI‐based tumor delineation. Overall, the similarity of average GTV delineation between T2w 4DMRI and 4DCT provides support for the clinical application of T2w 4DMRI to delineate lung tumors.

Patient breathing irregularities are common, causing various known issues, such as binning artifacts for GTV target delineation and tumor motion variations for ITV delineation. The GTV variation within the breathing cycle was reported to be as large as 110%^35^, ^36^ and observed as 109% in this study. The GTV motion variation was reported up to 200% from 1cm motion in 4DCT (simulation) and 3 cm motion in fluoroscopy (treatment).^37^ In this study, the GTV variation is as large as 109% and ITV variation is (−25 to 95%) between 4DCT and 4DMRI, consistent with the previous finding.

## 5 CONCLUSION

The feasibility of using 4DMRI for GTV and ITV delineation of lung cancer in radiotherapy has been demonstrated by comparison with 4DCT. The mean GTV from T2w‐based (97%) is similar to CT‐based GTV (100%) while the T1w‐based GTV is 24% smaller (76%). This trend is more consistent for small/medium peripheral (detached) lung tumors. The average relative inter‐observer variation is increasing from T2w 4DMRI (5%), to 4DCT (14%) and T1w BH MRI (25%), suggesting a higher agreement among physicians when using T2w 4DMRI. Due to breathing irregularities, a large ITV variation (−25% to 95%) between 4DMRI and 4DCT is observed, implying a variation between simulation and treatment. It is necessary to reduce the intra‐ and inter‐observer variation by further MRI (T2w and T1w) training for tumor delineation.

## CONFLICT OF INTERESTS

There is a master research agreement between Memorial Sloan Kettering Cancer Center and Philips Healthcare and this research uses the respiratory‐correlated (RC) 4DMRI acquisition software for patient data acquisition. Some co‐authors have various grant and nongrant supports, which are not directly related to this research.
